# Health impacts of the July 2010 heat wave in Québec, Canada

**DOI:** 10.1186/1471-2458-13-56

**Published:** 2013-01-21

**Authors:** Ray Bustinza, Germain Lebel, Pierre Gosselin, Diane Bélanger, Fateh Chebana

**Affiliations:** 1 Institut national de santé publique du Québec, Direction de la santé environnementale et de la toxicologie, Québec, Canada; 2 Université Laval, Québec, Canada; 3 Centre hospitalier universitaire de Québec, Centre de recherche, Québec, Canada; 4 Institut national de la recherche scientifique, Centre eau-terre-environnement, Québec, Canada

## Abstract

**Background:**

One of the consequences of climate change is the increased frequency and intensity of heat waves which can cause serious health impacts. In Québec, July 2010 was marked by an unprecedented heat wave in recent history. The purpose of this study is to estimate certain health impacts of this heat wave.

**Methods:**

The crude daily death and emergency department admission rates during the heat wave were analyzed in relation to comparison periods using 95% confidence intervals.

**Results:**

During the heat wave, the crude daily rates showed a significant increase of 33% for deaths and 4% for emergency department admissions in relation to comparison periods. No displacement of mortality was observed over a 60-day horizon.

**Conclusions:**

The all-cause death indicator seems to be sufficiently sensitive and specific for surveillance of exceedences of critical temperature thresholds, which makes it useful for a heat health-watch system. Many public health actions combined with the increased use of air conditioning in recent decades have contributed to a marked reduction in mortality during heat waves. However, an important residual risk remains, which needs to be more vigorously addressed by public health authorities in light of the expected increase in the frequency and severity of heat waves and the aging of the population.

## Background

One of the most indisputable consequences of climate change is the increased frequency and intensity of heat waves [1]. A heat wave can have particularly serious health impacts, even in the context of developed countries. In three weeks, the number of deaths due to the 2003 heat wave in eight European countries was close to 35,000 people [2]. In the United States, the heat wave that hit California for two weeks in 2006 led to an excess of at least 140 deaths, but also 16,166 emergency department admissions and 1,182 hospitalizations [3]. In Canada, 2010 was the hottest year and summer ever recorded in 63 years of meteorological observations [4]. For Ontario and Québec, July 2010 was marked by an unprecedented heat wave from July 4^th^ to 9^th^, with maximum temperatures reaching 30°C or more, minimum temperatures above 20°C, and high humidity. The purpose of this study is to estimate certain health impacts of the July 2010 heat wave in Québec. More precisely, this study describes the number of deaths and emergency room admissions related to the heat wave, and discusses how this could be explained compared to previous similar episodes.

## Methods

### Sources of data

The meteorological data are from Environment Canada. In this study, they include the minimum and maximum daily temperatures observed at the reference weather stations located within health regions (HRs). A reference station is a station considered as representative of a given region by Environment Canada [5]. The number of deaths (all causes) was extracted from three databases of the *Institut de la Statistique du Québec*: daily files (2010), provisional files (2008–2009), and final files (2005–2007); provisional files are usually quite similar to final files except for a few changes in final cause of death (no impact for this study as all-cause is used) and the addition of deaths occurring out of province, which could result in a very slight underestimation of the daily death rates here. In the daily files database, age is available only according to three strata: < 65, 65–74, and > 74. The number of emergency department admissions (all causes) comes from the *Relevé quotidien de la situation dans les urgences et les centres hospitaliers* (RQSUCH, daily report of the situation in emergency departments and hospitals) of the *ministère de la Santé et des Services sociaux du Québec* (Quebec ministry of Health and Social Services), just like the population numbers taken from demographic projections [6]. All data used in this study is openly available.

### Analysis periods

The heat wave period has been defined as the days in July 2010 when the moving averages (over three days) of the minimum and maximum temperatures of the HRs were equal to or exceeded certain predefined thresholds (Table 1). These thresholds, established previously [5,7] in 16 of the 18 HRs of Québec, were defined in such a way as to predict the hot periods that could produce significant excesses in fatalities of 60% or more, compared to the 1981–2005 historical daily average. The level of 60% was used because of the low number of daily deaths in historical data per HR, in order to increase the statistical stability of thresholds and decrease the number of false alerts in forecasts.

**Table 1:** Characteristics of the heat wave, by health region (HR)

**HR**	**Date of start**	**Total duration (days)**	**Maximum temperatures**			**Minimum temperatures**		
			**Thresholds (°C)**	**Highest**		**Thresholds (°C)**	**Highest**	
				**°C**	**Number of days after the start**		**°C**	**Number of days after the start**
Capitale-Nationale	05/07	5	31.0	33.4	2	16.0	21.0	1
Chaudière-Appalaches	05/07	5	31.0	33.5	2	16.0	21.2	3
Estrie	05/07	5	31.0	33.3	3	18.0	22.2	3
Lanaudière	05/07	5	33.0	34.8	3	20.0	22.8	2
Laval	05/07	4	33.0	33.7	3	20.0	25.5	1
Montérégie	05/07	4	33.0	34.7	3	20.0	24.0	1
Montréal	05/07	4	33.0	33.7	3	20.0	25.5	1
Outaouais	04/07	5	31.0	34.3	4	18.0	20.3	2

To estimate the health impacts, the period of interest corresponded to the heat wave plus the following three days (hereafter called the impact period). The three days are added to take into account the delayed effects of heat on health and are based on the known duration of the impacts of heat on all-cause mortality [8-12]. To evaluate a potential mortality displacement (also known as harvesting effect), the total mortality in the 60 days (without a heat wave) after the impact period was also studied. The comparison periods met the following criteria: 1) corresponded to the same days of the week during the years 2005, 2006, 2007, 2008, and 2009; 2) corresponded to the dates closest to the 2010 impact period; and 3) did not include any heat wave.

### Statistical analyses

The crude death and emergency department admission rates for the impact period were analyzed in relation to the comparison periods using 95% confidence intervals [13]. Since the crude rate follows a Poisson distribution, the method used to calculate confidence intervals for crude rate is the normal approximation of the natural logarithm of the rate [14].

## Results

### Characteristics of the heat wave

In July 2010, eight of the 18 HRs of Québec experienced a heat wave lasting an average of 4.6 days (range: 4 to 5 days). The highest maximum temperatures varied from 33.3 to 34.8°C and were observed for 2.9 days on average (range: 2 to 4 days) after the start of the heat wave, whereas the highest minimum temperatures fluctuated between 20.3 and 25.5°C and were observed for 1.8 days on average (range: 1 to 3 days) after the start (Table 1). These values, just like the averages, were clearly higher in July 2010 (compared to 2005–2009) (Table 2).

**Table 2:** **Average temperatures during the heat wave (2010) and the comparison periods (2005*–*2009), by health region (HR)**

**HR**	**Maximum temperatures (°C)**		**Minimum temperatures (°C)**	
	**2010**	**2005-2009**	**2010**	**2005-2009**
Capitale-Nationale	32.3	24.0	19.7	12.8
Chaudière-Appalaches	32.1	23.6	19.8	11.2
Estrie	32.3	24.8	21.0	12.5
Lanaudière	33.6	25.3	21.2	13.6
Laval	33.4	24.8	24.0	15.8
Montérégie	34.0	24.7	21.6	14.0
Montréal	33.4	24.8	24.0	15.8
Outaouais	33.1	23.9	18.5	12.4

### Health impacts

**Figure 1:**
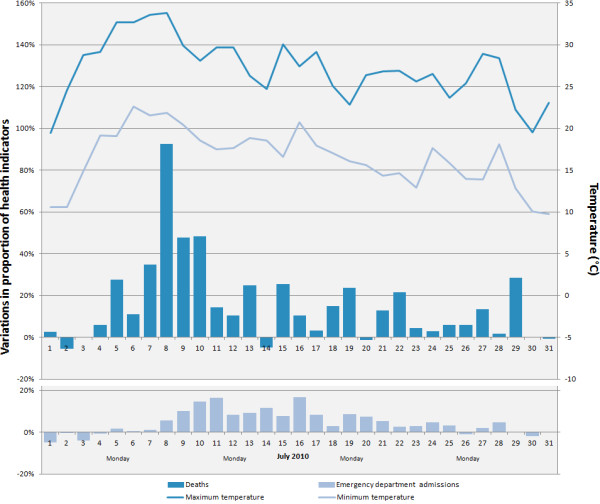
Evolution of daily variations in proportion of health indicators in all of the eight RSSs of Quebec affected by the heat waves from July 1st to 31st, 2010 versus the comparison periods (2005-2009) and daily averages of July 2010 temperatures.

Collectively, the eight HRs affected by the heat wave (approximately six million people) showed a significant increase of 33% in the crude death rate (about 280 extra deaths) and 4% in the crude emergency department admission rate (about 3400 extra admissions), compared to 2005–2009 (Table 3). The 2010 crude rates by age group showed a significant increase of about 33% compared to 2005–2009 for the 0–64 year and 75 year or older group; the 65–74 year group showed a non-significant increase (Table 4). Four of the eight HRs (Capitale Nationale, Chaudière-Appalaches, Laval and Montréal) had higher emergency department admission rates, and three (Montréal, Montérégie and the Outaouais) had higher death rates (Table 3). The deaths show a 93% maximum daily increase on the fourth day of the heat wave, and the emergency department admissions, a 17% increase on the seventh day of the period (Figure 1).

**Table 3:** Crude death and emergency department admission rates, by health region (HR) and study period

**HR (population 2010)**	**Period**	**Deaths**		**Emergency dept. admissions**	
		n*	**Crude rates per 100,000 person-days (95% CI)**	n*	**Crude rates per 100,000 person-days (95% CI)**
All HRs (5 985 001)	2005-2009	760	1.77 (1.71–1.83)	47 635	110.89 (110.45–111.34)
	2010	1 039	**2.35 (2.22**–**2.50)**	51 054	**115.70 (114.70–116.70)**
Capitale Nationale (687 950)	2005-2009	107	1.98 (1.82–2.16)	9 219	171.09 (169.54–172.66)
	2010	114	2.07 (1.72–2.49)	9 704	**176.32 (172.85–179.86)**
Chaudière-Appalaches (405 576)	2005-2009	58	1.81 (1.61–2.03)	3 676	115.17 (113.52–116.85)
	2010	53	1.63 (1.25–2.14)	5 058	**155.89 (151.65–160.25)**
Estrie (308 335)	2005-2009	47	1.96 (1.72–2.22)	4 032	166.45 (164.17–168.77)
	2010	54	2.19 (1.68–2.86)	4 080	165.40 (160.41–170.56)
Lanaudière (468 381)	2005-2009	55	1.56 (1.39–1.76)	2 308	65.24 (64.06–66.44)
	2010	77	2.05 (1.64–2.57)	2 425	64.72 (62.19–67.35)
Laval (396 186)	2005-2009	42	1.58 (1.38–1.81)	1 264	47.70 (46.54–48.89)
	2010	62	2.24 (1.74–2.87)	1 500	**54.09 (51.42–56.89)**
Montérégie (1 444 047)	2005-2009	165	1.69 (1.58–1.81)	7 889	80.65 (79.85–81.45)
	2010	233	**2.31 (2.03–2.62)**	8 299	82.10 (80.35–83.89)
Montréal (1 912 388)	2005-2009	240	1.82 (1.72–1.93)	15 498	117.50 (116.67–118.33)
	2010	376	**2.81 (2.54**–**3.11)**	16 233	**121.19 (119.34–123.07)**
Outaouais (362 138)	2005-2009	46	1.63 (1.43–1.86)	3 747	134.24 (132.33–136.17)
	2010	70	**2.42 (1.91–3.05)**	3 765	129.96 (125.87–134.18)

The 2010 rates, statistically different from the comparison rates (2005–2009) based on the confidence intervals (CI), are shown in bold.

* Average numbers for the 2005–2009 period.

**Table 4:** Crude death rates, by age and study period for all regions affected

**Age group**	**Period**	**Deaths**	
		n*	**Crude rates per 100,000 person-days (95% CI)**
0-64 years	2005-2009	178	0.49 (0.46–0.52)
	2010	240	**0.65 (0.58**–0**.74)**
65-74 years	2005-2009	132	3.99 (3.70–4.31)
	2010	160	4.40 (3.77–5.14)
75+ years	2005-2009	450	15.99 (15.34–16.66)
	2010	639	**21.22 (19.64–22.93)**

The 2010 rates, statistically different from the comparison rates (2005–2009) based on the confidence intervals (CI), are shown in bold.

* Average numbers for the 2005–2009 period.

### Mortality displacement

Mortality displacement (or harvesting effect) could not be studied in three of the eight HRs affected by the July 2010 heat wave due to the presence of other heat episodes in the 60 days following the impact period. In the five other HRs (Lanaudière, Laval, Montérégie, Montréal and Québec; about 5 million people), crude daily death rates comparable to those reported in 2005–2009 were observed over the following 60-day period (Table 5).

**Table 5:** Crude death rates during the 60 days following the 2010 heat waves, by health region (HR) and study period

**HR (2010 population)**	**Period**	**Deaths**	
		n*	**Crude rates per 100,000 person-days (95% CI)**
All HRs (4 908 952)	2005-2009	5 189	1.81 (1.79–1.83)
	2010	5 397	1.83 (1.78–1.88)
Capitale Nationale (687 950)	2005-2009	805	1.99 (1.93–2.05)
	2010	804	1.95 (1.82–2.09)
Lanaudière (468 381)	2005-2009	431	1.62 (1.56–1.69)
	2010	436	1.55 (1.41–1.70)
Laval (396 186)	2005-2009	370	1.63 (1.56–1.71)
	2010	428	1.80 (1.64–1.98)
Montérégie (1 444 047)	2005-2009	1 390	1.66 (1.62–1.70)
	2010	1 483	1.71 (1.63–1.80)
Montréal (1 912 388)	2005-2009	2 193	1.94 (1.90–1.98)
	2010	2 246	1.96 (1.88–2.04)

CI = confidence intervals.

* Average numbers for the 2005–2009 period.

## Discussion

Collectively, the eight Québec HRs affected by the July 2010 heat wave showed significant increases in the crude death and emergency department admission rates in relation to the comparison periods. However, the regional analysis identified important variations. Only three HRs had a significant increase in crude emergency department admission rates, and only the Montréal health region had higher emergency department admission and death rates simultaneously. Finally, no mortality displacement was observed over a 60-day horizon.

### Deaths

As elsewhere worldwide, this study shows that heat waves can be fatal weather events [15-18]: the significant increase (33%) in crude rate is comparable to the results reported elsewhere [16,19]. However, it is lower than during two similar episodes that occurred in the Montréal HR in 1987 and 1994 when the daily mortality exceeded 100% of the historical average [20]. At the time of these previous episodes, no public health intervention plan existed and no preventive measures had been implemented. Since 2006, a formal Heat Action Plan [21] has been implemented at the provincial level for all relevant HRs, promoting a proactive approach. Moreover, the new thresholds proposed [5,7], although based on a 60% excess mortality of historical heat wave episodes, were used to establish forecast thresholds for full public health intervention before the heat wave, or at its very beginning. Since we know that heat-related deaths are largely preventable through appropriate communication and prevention [22], this more organized and proactive approach could partly explain the lower excess mortality, even with an aging population. This situation could also be partly explained by the gradual changes in the population's behaviour during heat waves, notably the increased use of air conditioning systems. Since 1987, for all households in the province of Québec, the ownership of air conditioning systems increased from 15% to 42% in 2010 [23,24]. However, the actual use of air conditioning is not measured and will vary significantly according to income, even during heat waves [25]. Unfortunately, none of this data is available by HR, which could have helped greatly in the interpretation of some of our results.

This study also shows that heat-related mortality increases rapidly from the start of the heat wave [8-11], and some of its health impacts are detectable up to three days after the maximum temperature peak [12,26]. Thus, some regional differences in heat exposure (and occurrence/ absence of rain) could explain the regional variability. The HRs of Montréal and Montérégie are the most populous in Québec, and contain significant urban heat island areas which promote an increased exposure of the population to heat [12,22,27,28]. Also, the characteristics of the heat wave are different from one region to another (Table 1). The highest maximum temperatures (≥ 34°C) involve only three HRs, including those of Outaouais and Montérégie. Otherwise, the highest minimum temperatures (≥ 24°C) involve only three HRs, including those of Montréal and Montérégie. In these highly urbanized regions, the minimum temperature peaks were reached very rapidly, namely 24 hours after the start of the heat wave. It seems that the maximum value of the minimum temperature and the time to reach this peak are important factors in estimating the intensity of a heat wave and the severity of its impacts [29,30].

The lack of statistically different impacts in the HRs other than Montréal, Outaouais and Montérégie could also be due to the small numbers of health events in the less populated regions and to the related low power of detection, or even to differences related to certain individual parameters such as age [16,19,22] or health status [12,31-34], but we have no evidence to this effect. Other studies would be needed to clarify these aspects.

The graph (Figure 1) of the daily variations (2010 vs. 2005–2009) of the all-cause deaths between July 1 and 31, 2010, suggests that this indicator has several qualities that make it useful in watching for and monitoring heat waves. In fact, there is a short period (a few hours) between the start of the heat wave and the increase in deaths, and deaths increase right from the first day. The all-cause death indicator seems to be sufficiently sensitive and specific to exceedences of the temperature thresholds, because the daily variations in deaths clearly reflect the fluctuations in temperatures during the heat wave. In this case, analysis of mortality over a 60-day horizon after the heat wave does not show any significant lower mortality, contrary to some other studies [35-38]. For some heat waves, a decrease in mortality was observed during the weeks after the wave. This short-term forward shift in mortality is also referred to as mortality displacement, or harvesting effect. This reduction in mortality usually suggests that the heat wave particularly affected individuals whose health is already so compromised that they would have died in the short term anyway [39]. Hence, in the absence of mortality displacement, it seems that the deaths measured in this study were primarily due to the July 2010 heat wave, and not to the early mortality of weakened individuals. On the other hand, we may not have detected lower forward mortality because of our methodology (e.g., periods chosen for studying the delayed effects) or the characteristics of our studied populations compared to other studies [40].

Finally, our results do not reveal a greater increase in deaths in the elderly (75 years of age and older) compared to the 0–64 year group as in other studies [22,30,41-43]. The Québec population may have various strategies for adapting to heat, independent of their age (e.g., heat wave warnings issued in the regions involved, and action plans targeting the elderly). Nonetheless, it is also possible that this absence of a greater increase in deaths in the elderly could be explained by the coarse nature of the variable (in the Québec daily death record, age is classified according to three strata only: < 65, 65–74, and > 74).

### Emergency department admissions

In this study, the significant increase in emergency department admissions (4%) for all of the HRs affected by the July 2010 heat wave is similar to the increase recently observed in a California study (3%) [3]. In addition, there are some rate variations across the HRs that could be explained by local factors, but we do not have enough information to reach any conclusion about the reasons for these variations. Furthermore, the graph (Figure 1) of the daily variations in emergency department admissions (2010 vs. 2005–2009) indicates that they are modest and do not reflect the fluctuations in temperature. Such modest variations in the impact of a heat wave on these daily variations were also reported in a French study in 2005 [44]. Based on this information, the usefulness of this indicator for monitoring the health impact of a heat wave is not as clear as in the case of deaths. Nevertheless, this indicator remains useful for hospital management in such a context.

### Limitations

Our study is based on the analysis of a single heat episode, which limits the generalization of results. The study is also affected by the difficulty in characterizing exposure in ecological studies. As well, the temperature values come from a single reference weather station per HR, even though each HR generally includes several cities. However, even if there could be some variation in temperatures in different parts of a region, the reference station reflects well the temperatures of the most populated areas, and thus provides valid temperatures for most of the population, according to the definition of a reference station by Environment Canada. Additionally, the presence of heat islands (and associated risk) is also greater in the more urbanized areas.

It should be mentioned that the temporary death file contains only coarse information about age and no information about the diagnosis, which limits further data interpretation. Finally, the analysis did not take atmospheric pollutants into account. This could have explained some regional differences regarding the impact on mortality, but this remains a hypothesis since the effect of atmospheric pollution on the temperature-mortality relationship remains highly controversial [15,27,40,45].

## Conclusions

In Québec, public health and civil-preparedness intervention during heat wave episodes is based on a common intervention guide developed in 2004 and updated in 2006 [21], on Environment Canada weather forecasts, on specific intervention thresholds by regions, and on the *Système de surveillance et de prévention des impacts sanitaires des évènements météorologiques extrêmes* (SUPREME, system for surveillance and prevention of the health impacts of extreme weather events) [46,47]. This system, developed by the *Institut national de santé publique du Québec*, has been available since May 2010 for the province’s regional and central public health authorities. Based on weather forecasts and population vulnerability indicators, heat wave warnings can be issued and interventions implemented to prevent various health impacts in such a context. These actions, combined with major advancements in air conditioning in the last 20 years, have contributed to a marked reduction in mortality during heat waves, even in the presence of a doubling of the proportion of the elderly during the same period of time. Important residual risk remains, however, which needs to be more vigorously addressed by public health authorities in light of the expected increase in frequency and severity of heat waves and the expected further aging of the population.

## Competing interests

No competing interest is declared. This research was funded by the Green Fund in the framework of Action 21 of the Québec government’s 2006–2012 Climate Change Action Plan.

## Authors’ contributions

RB and GL were the principal researchers and authors. PG, DB and FC were co-authors and co-investigators. All authors read and approved the final manuscript.

## Pre-publication history

The pre-publication history for this paper can be accessed here:

http://www.biomedcentral.com/1471-2458/13/56/prepub
